# Structural and functional characterization of nanobodies that neutralize Omicron variants of SARS-CoV-2

**DOI:** 10.1098/rsob.230252

**Published:** 2024-06-05

**Authors:** Katy Cornish, Jiandong Huo, Luke Jones, Parul Sharma, Joseph W. Thrush, Sahar Abdelkarim, Anja Kipar, Siva Ramadurai, Miriam Weckener, Halina Mikolajek, Sai Liu, Imogen Buckle, Eleanor Bentley, Adam Kirby, Ximeng Han, Stephen M. Laidlaw, Michelle Hill, Lauren Eyssen, Chelsea Norman, Audrey Le Bas, John Clarke, William James, James P. Stewart, Miles Carroll, James H. Naismith, Raymond J. Owens

**Affiliations:** ^1^ Structural Biology, The Rosalind Franklin Institute, Harwell Science Campus, Didcot, UK; ^2^ Division of Structural Biology, Nuffield Department of Medicine, University of Oxford, Oxford, UK; ^3^ Nuffield Department of Medicine, Pandemic Sciences Institute, University of Oxford, Oxford, UK; ^4^ Wellcome Centre for Human Genetics, University of Oxford, Oxford, UK; ^5^ Department of Infection Biology & Microbiomes, Institute of Infection, Veterinary and Ecological Sciences, University of Liverpool, Liverpool, UK; ^6^ Vetsuisse Faculty, Laboratory for Animal Model Pathology, Institute of Veterinary Pathology, University of Zurich, Zurich, Switzerland; ^7^ Diamond Light Source Ltd, Harwell Science Campus, Didcot, UK; ^8^ James & Lillian Martin Centre, Sir William Dunn School of Pathology, University of Oxford, Oxford, UK

**Keywords:** nanobodies, Omicrons, spike protein, SARS-CoV-2, COVID-19

## Abstract

The Omicron strains of SARS-CoV-2 pose a significant challenge to the development of effective antibody-based treatments as immune evasion has compromised most available immune therapeutics. Therefore, in the ‘arms race’ with the virus, there is a continuing need to identify new biologics for the prevention or treatment of SARS-CoV-2 infections. Here, we report the isolation of nanobodies that bind to the Omicron BA.1 spike protein by screening nanobody phage display libraries previously generated from llamas immunized with either the Wuhan or Beta spike proteins. The structure and binding properties of three of these nanobodies (A8, H6 and B5-5) have been characterized in detail providing insight into their binding epitopes on the Omicron spike protein. Trimeric versions of H6 and B5-5 neutralized the SARS-CoV-2 variant of concern BA.5 both *in vitro* and in the hamster model of COVID-19 following nasal administration. Thus, either alone or in combination could serve as starting points for the development of new anti-viral immunotherapeutics.

## 1. Introduction

Although vaccination programmes have been successful in combatting the SARS-CoV-2 pandemic, the emergence of new variants continues to undermine immune protection, especially in immunocomprised individuals. Immunization with spike protein vaccines based on the original Wuhan strain has proved much less effective for protection against subsequent variants and in particular the Beta (B.1.351) [1,2] and, more recently, Omicron strains of SARS-CoV-2 (B.1.1.529) [3–6]. A similar picture has been observed for the therapeutic monoclonal antibodies that were derived from patients originally infected in the first wave of the pandemic [7].

The Beta variant was the first to contain the mutation E484K in the receptor-binding domain (RBD) of the spike protein that disrupted the binding interactions of many antibodies [2], with a second mutation, N501Y, which also occurs in the Delta variant (B.1.617.2), contributing to loss of binding activity. The Omicron variants feature the most changes from the prototypical Wuhan sequence that have been found so far with six changes in the RBD, including E484A and N501Y [8], disrupting the epitopes of many but not all human antibodies to the virus [5].

Vaccines based on variant sequences will be deployed but there remain the issues that some individuals respond poorly to vaccination, some are not vaccinated and further variants may arise. Therefore, the development of broadly cross-reactive antibodies that can be used in passive immunotherapy or prophylactic treatments remains an active area of research and development.

The immunodominant epitopes of the spike protein are localized in the RBD and detailed structural analyses of antibody–RBD complexes have enabled the binding profiles to different SARS-CoV-2 variants to be rationalized. The structural knowledge coupled to genetic sequencing of the variants provides a basis for identifying potentially cross-reactive binders. In addition to human-derived monoclonal antibodies, a feature of the pandemic has been the isolation of a number of single-domain antibodies (nanobodies) against SARS-CoV-2 spike protein that show potent neutralizing SARS-CoV-2 activity *in vitro* [1,9–16] and in animal models of COVID-19 [1,15,17–20]. Structural analyses have mapped out the epitopes of these nanobodies on the RBD and have shown that the majority cluster in two regions: one on the side of the RBD, distal from the human angiotensin converting enzyme-2 (ACE-2) receptor binding interface (cluster 1), while the second are close to or at the ACE-2 binding region (cluster 2) [21]. These categories correspond to the Class 4 and Class 1/2 nomenclature proposed for human anti-SARS-CoV-2 antibodies [22]. Recently, analysis of a large panel of llama-derived nanobodies to the RBD has identified a further three binding classes and a direct correlation between virus neutralization potency and the distance between where nanobodies and ACE-2 bind to the RBD [23]. In most examples, effective virus neutralization has been achieved by an assembly of nanobodies into multivalent molecules, for example, by genetically fusing to an immunoglobulin Fc fragment or joining one or more nanobodies together (head to tail) that bind to the same (homopolymers) or different (heteropolymers) epitopes on the RBD. The design of hetero-nanobody polymers, so-called bi-paratopic binders, has been informed by the structures of the component nanobodies in complex with the RBD [11,24].

We previously reported the generation of several nanobodies raised to the Wuhan virus spike protein that mapped to clusters 1 and 2 binding regions [1]. Trimeric versions of these nanobodies showed potent neutralization activity against the original Wuhan virus and the two SARS-CoV-2 variants of concern (VOCs) prevalent in early 2021 (Beta B.1.351 and Alpha B.1.1.7). Here, we report the isolation and characterization of new cross-reactive nanobodies that, configured as trimers, potently neutralize the Omicron virus as well as the other major viral variants. The nanobodies were identified by re-screening our existing phage display libraries generated from a llama immunized with the Wuhan and Beta spike protein, showing how nanobodies to future SARS-CoV-2 VOCs could be rapidly generated.

## 2. Results

### 2.1. Selection and characterization of nanobodies to SARS-CoV-2 RBD variants

We previously reported the construction of a nanobody library from a llama (named Fifi) immunized with the Wuhan spike protein [1]. To broaden the repertoire of llama-derived variable heavy heavies (VHHs), a second library was constructed from the same llama immunized with the Beta spike protein. We reasoned that this would generate antibodies that tolerated the key mutations at residues E484K/A and N501Y in the RBD of the spike protein found in both Beta and subsequently Omicron variants. Both the Wuhan and Beta libraries were screened with the Beta RBD from which two new nanobodies were isolated, designated A8 (Wuhan library) and H6 (Beta library). These nanobodies were selected from the results of phage ELISA and sequencing (electronic supplementary material, figure S1 and table S2). The monomeric A8 and H6 nanobodies were produced in *Escherichia coli* and their binding affinities for the Beta RBD measured by biolayer interferometry [25]. The results confirmed that both nanobodies bound strongly to this RBD with affinities of 0.89 and 0.68 nM, respectively (table 1; electronic supplementary material, figure S2).

**Table 1.** Kinetic binding affinities of monomeric nanobodies for the RBDs of SARS-CoV-2 measured by biolayer interferometry [25].

nanobody	antigen	average *K*_D_ (nM)	average *k*_a_ (1/Ms)	average *k*_dis_ (1/s)
A8 monomer	Beta RBD	0.889	3.26E+05	2.89E−04
	BA.1 RBD	15.7	3.43E+05	5.30E−03
	BA.4/5 RBD	8.10	3.49E+05	2.83E−03
H6 monomer	Beta RBD	0.677	4.98E+05	3.37E−04
	BA.1 RBD	22.7	2.45E+05	5.53E−03
	BA.4/5 RBD	7.08	3.33E+05	2.35E−03
B5-5 monomer	Beta RBD	0.0655	2.16E+05	1.29E−05
	BA.1 RBD	0.355	5.76E+05	2.04E−04
	BA.4/5 RBD	0.684	1.90E+05	1.29E−04
A10-5 monomer	Beta RBD	<1.0 pM	1.78E+05	<1.0E−07
	BA.1 RBD	<1.0 pM	7.55E+04	<1.0E−07
	BA.4/5 RBD	<1.0 pM	8.09E+04	<1.0E−07
A3-8 monomer	BA.1 RBD	0.0258	3.52E+05	9.09E−06
	BA.4/5 RBD	424	4.22E+05	0.179
A4-8 monomer	BA.1 RBD	1.25	5.68E+05	2.03E−04
	BA.4/5 RBD	6.17	2.03E+05	1.25E−03

With the subsequent emergence of the Omicron variants, both the Wuhan and Beta VHH libraries were re-screened with the stabilized spike trimer of the BA.1 Omicron subvariant [5] and a set of four new binders selected for further characterization: A3-8, A4-8, A10-5 and B5-5. Together with nanobodies A8 and H6, the affinities of these four nanobodies for the RBDs from Omicron subvariants BA.1 and BA.4/5 were measured by BioLayer Interferometry (BLI). The results are summarized in table 1 and representative sensorgrams shown for A8, H6 and B5-5 in electronic supplementary material, figure S2. All five nanobodies bound to the Omicron BA.1 RBD with high affinity, with those directly selected by panning with the BA.1 spike protein showing the lowest *K*_D_ values, particularly A10-5, the affinity of which could not be accurately measured below picomolar. Except for nanobody A3-8, the others showed comparable binding affinities to the RBD of BA.4/5 (table 1; electronic supplementary material, figure S2). These results indicated that the five nanobodies are likely to recognize distinct epitopes.

To identify the relative location of the nanobodies within the BA.1 RBD, epitope binning experiments were carried out using BLI. Briefly, sensors pre-coated with biotinylated BA.1 RBD were loaded with one nanobody, washed and then the association and dissociation kinetics were measured for a second nanobody. From the results, we concluded that B5-5, A3-8 and A10-5 bound to epitopes that overlapped whereas H6, A8 and A4-8 each bound to a distinct epitope that did not overlap with the other epitopes (electronic supplementary material, figure S3).

**Figure 1.**
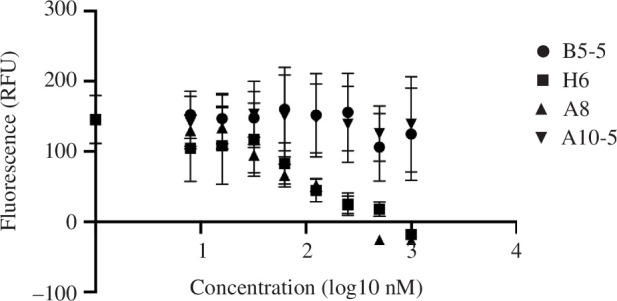
Inhibition of SARS-CoV-2 spike binding to cell-expressed ACE-2 by monomeric nanobodies. Quantitation of the inhibition of SARS-CoV-2 Omicron BA.1 trimers binding to ACE-2 expressed on Calu3 cells, by incubation with nanobody monomers (A8, H6, B5-5 and A10-5). The spike protein was labelled with biotin-Alexafluor568 via a C-terminal Avitag. The Calu3 cells stably expressed full-length human ACE-2 fused to GFP protein. The data are shown as the mean (*n* = 3) ± s.e.

Binding of the SARS-CoV-2 spike protein to the human ACE-2 receptor at the cell surface is the first step in viral entry and blocking this interaction correlates with neutralization of infection [26]. A cell-based assay was established in which the binding of fluorescently labelled stabilized BA.1 spike trimer to Calu3 cells stably expressing GFP-tagged ACE-2 in the membrane is visualized by confocal microscopy. Pre-incubation of the spike protein with either A8 or H6 led to a nanobody concentration-dependent inhibition of spike binding to Calu3 cells. By contrast, neither B5-5 nor A10-5 inhibited spike trimer binding to the cells and B5-5 (figure 1; electronic supplementary material, figure S4).

### 2.2. Structural analysis of nanobody–spike and nanobody–RBD complexes

**Figure 2.**
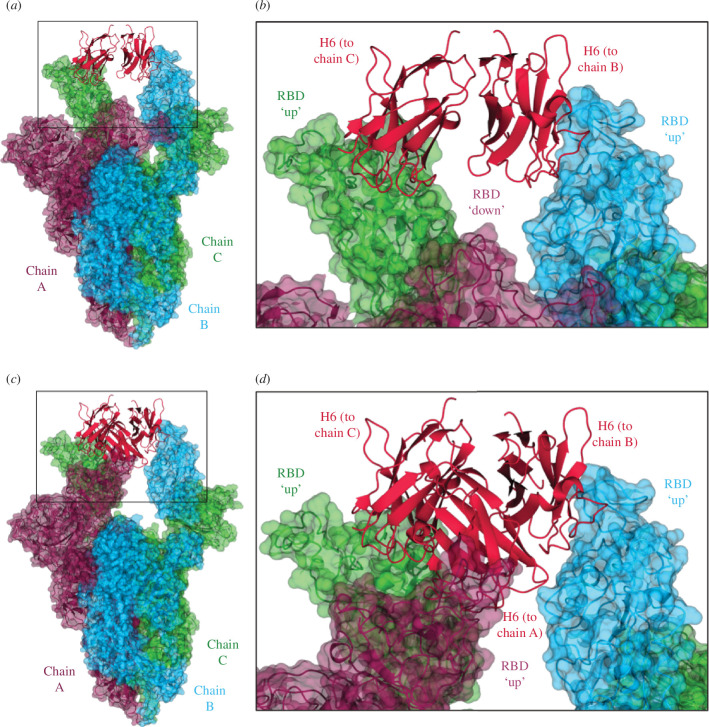
EM data of spike–Nb complexes. (*a*) EM structure of stabilized hexaPro Omicron BA.1 spike trimer in the two RBD ‘up’ and one RBD ‘down’ conformation with two H6 nanobodies bound. H6 monomers are shown in crimson, and the spike monomers are coloured dark purple (chain A), sky blue (chain B) and lime green (chain C), with chain A in the RBD ‘down’ conformation, and chains B and C in the RBD ‘up’ conformation. (*b*) Close-up of the boxed region from (*a*) showing the binding of the H6 nanobodies to the ‘up’ RBDs of chains B and C, with no nanobody bound to the ‘down’ RBD of chain A. (*c*) EM structure of stabilized hexaPro Omicron BA.1 spike trimer in the three RBD ‘up’ conformation with three H6 nanobodies bound. (*d*) Close-up of the boxed region from (*a*) showing the binding of an H6 monomer to the ‘up’ RBD of each chain. Figures were generated using CCP4mg [28].

Nanobodies A8, H6 and B5-5 were selected for further investigation of their relative binding sites by structural analysis of nanobody–spike/RBD complexes. The monomeric nanobodies were each mixed with a hexaPro stabilized version of the BA.1 spike protein trimer in a 3.6:1 molar ratio and then vitrified on cryo-EM grids. The incorporation of six prolines in the S2 domain has been shown to considerably increase the stability of this version of the prefusion trimer [27]. However, incubation with nanobodies A8 and B5-5 appeared to disrupt the assembly of the spike proteins as no trimers were observed on the EM grids. We have observed this behaviour previously for nanobodies C1 and F2(1) and we speculate that A8 and B5-5 may bind to sites on the spike protein that are not compatible with trimer assembly. By contrast, H6 formed a complex with the BA.1 spike trimer and an approximately equal mixture of the two forms was observed on the grids (figure 2; electronic supplementary material, figure S5; table 2). In one, an H6 nanobody was bound to each RBD of the spike trimer in the so-called ‘all up’ conformation, and in the second the RBDs were in a ‘2 up 1 down’ state with two nanobodies bound to the ‘up’ conformation but missing from the ‘down’ conformation (figure 2). H6 is only able to bind the spike trimer in the ‘up’ conformation, with the epitope partly inaccessible when the RBD is ‘down’. In previous reports, and in contrast to other SARS-CoV-2 variants, the stabilized Omicron spike trimer has only been observed in a ‘1 up 2 down’ conformation in cryo-EM structures [29].

**Table 2.** Cryo-EM data collection, refinement and validation statistics.

	**s**pike-BAP **Wuhan + H6**	**s**pike **h**exaPro **Omicron + H6 3**Up	**s**pike **h**exaPro **Omicron + H6** 2Up1Down
data collection and processing			
magnification	81 000	120 000	120 000
voltage (kV)	300	200	200
electron exposure (e^−^/Å^2^)	50.1	40.5	40.5
defocus range (μm)	1.0–2.5	1.5–3.0	1.5–3.0
pixel size (Å/pix) (super-resolution)	1.072	1.2	1.2
symmetry imposed	C1	C1	C1
initial particle images (no.)	—	568 299	568 299
final particle images (no.)	—	104 001	131 621
map resolution (Å)	—	3.8	4
FSC threshold	—	0.143	0.143
map resolution range (Å)	—		
refinement^a^			
initial model used	—	7QO7	7QO7
model resolution (Å)	—	3.9	4.0
FSC threshold	—	0.143	0.143
model resolution range (Å)	—	2.9–5.3	3.1–6.2
map sharpening *B* factor (Å^2^)	—	−107	−145
model composition	—		
non-hydrogen atoms	—	29 170	28 223
protein residues	—	3674	3544
*B* factors (Å^2^)	—		
protein	—	400	352
r.m.s. deviations	—		
bond lengths (Å)	—	0.008	0.007
bond angles (°)	—	1.08	0.72
validation			
MolProbity score	—	2.2	2.04
clashscore	—	19.2	13
poor rotamers (%)	—	0.4	0.2
Ramachandran plot	—		
favoured (%)	—	93	94
allowed (%)	—	6.6	5.7
disallowed (%)	—	0.4	0.3

^a^ Nanobody excluded from refinement

We determined the crystal structures of the three nanobodies A8, H6 and B5-5 in complex with isolated RBDs to resolutions of 2.37, 1.73 and 1.97 Å, respectively (table 3). H6 was crystallized in complex with the Beta-RBD, whereas A8 and B5-5 were solved in complex with the Wuhan RBD. To obtain diffracting crystals of the A8 and H6 RBD complexes, a second nanobody was included in the crystallization trials, H3 and F2, respectively, that bind to orthogonal sites [1]. Inspection of the three structures confirmed that the epitopes recognized by the three nanobodies do not overlap and explained the observed inhibition of ACE-2.

**Figure 3.**
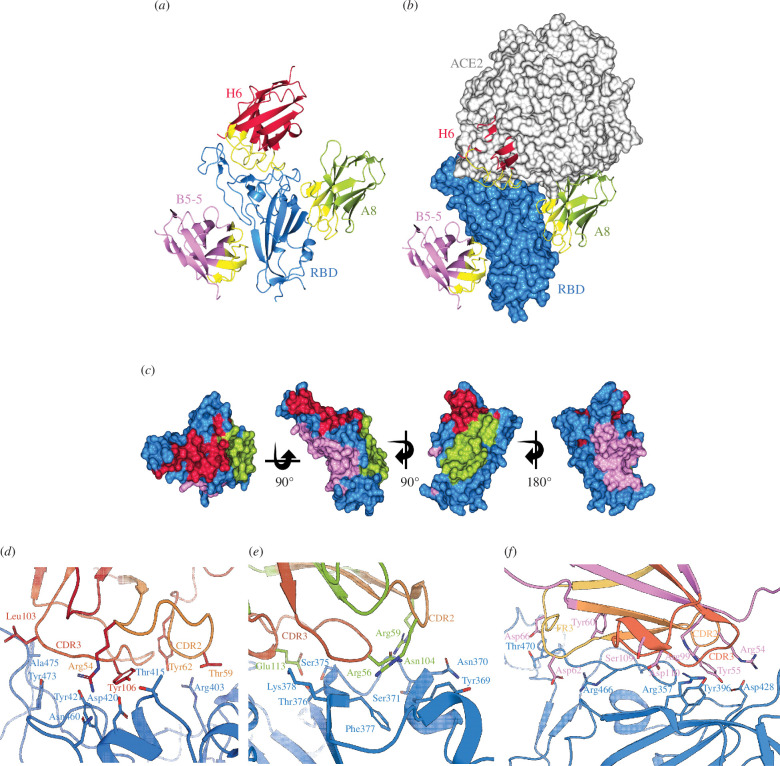
X-ray structures of nanobody–RBD complexes. (*a*) Ribbon diagram of the spike RBD (blue) with A8 (yellow-green) [30], H6 (crimson) and B5-5 (pink) nanobodies; CDR regions are shown in yellow. The figure was generated by superimposing the RBD protein from each crystal structure, with only one RBD monomer shown. (*b*) RBD is shown as a surface with the nanobodies coloured as in (*a*). Also shown is ACE-2 (pale grey surface) from the RBD–ACE-2 complex (PDB 6M0J), positioned by superposition of the RBD. (*c*) Surface representations showing the location of the binding locations of A8, H6 and B5-5 on the RBD, colour scheme as in (*a*). (*d*–*f*) Close up of the RBD–nanobody interfaces; for (*d*) A8–Wuhan RBD; (*d*–*f*) Close up of the RBD–nanobody interfaces; for (*d*) H6-Beta RBD; (*e*) A8–Wuhan RBD and ( *f*) B5-5-Wuhan RBD. Key interface residues are shown using stick representation. CDR2 (pale orange), FR3 (yellow) and CDR3 (brick orange) are indicated. Figures (*a*–*c*) were generated using CCP4mg [31], (*d*–*f*) using PyMOL (Schrödinger, LLC).

–RBD binding by A8 and H6 and the lack of inhibition by B5-5 (figure 3*a*,*b*). H6 binds directly at the ACE-2 receptor–RBD interface and completely occludes the site of ACE-2 binding and corresponds to Cluster 2(21) nanobodies (figure 3*a*,*b*). A8 binds on the side of the RBD at a similar location to other nanobodies including VHH72 [32] and C1(1) that sterically prevent ACE-2 binding (Cluster 1(21) nanobodies), whereas B5-5 binds on the opposite side of the RBD at a location that does not overlap with the ACE-2 binding region (figure 3*a*–*c*). By contrast to other regions of the RBD, there are only a few other nanobodies reported that bind to this face of the domain suggesting that it may be less immunogenic than other parts of the RBD [23,33–35].

**Table 3.** X-ray crystallography data collection and refinement statistics.

	A8-H3-RBD (8OWT)	H6-F2-RBD (8OWV)	B5-5-RBD (8OWW)
data collection			
space group	*P*2_1_2_1_2_1_	*P*2_1_2_1_2_1_	*P*22_1_2_1_
cell dimensions			
*a, b, c* (Å)	90.61, 97.20, 117.58	57.78, 59.44, 145.62	45.04, 70.03, 109.01
α, *β*, *γ* (°)	90, 90, 90	90, 90, 90	90, 90, 90
resolution (Å)^a^	58.78–2.14 (2.18–2.14)	59.52–1.73 (1.76–1.73)	45.04–1.97 (2.02–1.97)
*R*_merge_	0.309 (5.216)	0.105 (2.028)	0.201 (7.401)
*R*_pim_	0.088 (1.461)	0.031 (0.610)	0.028 (0.988)
*I*/σ (*I*)	6.5 (0.4)	11.5 (0.4)	15.3 (0.8)
*CC*_1/2_	0.996 (0.324)	0.999 (0.700)	0.999 (0.461)
completeness (%)	100.0 (100.0)	100.0 (100.0)	99.9 (99.0)
redundancy	13.7 (14.0)	12.5 (11.9)	53.6 (55.5)
refinement			
resolution (Å)^a^	58.78–2.37 (2.43–2.37)	59.52–1.73 (1.76–1.73)	45.04–1.97 (2.02–1.97)
no. reflections	42 863	53 238	25 113
*R*_work_/*R*_free_	0.213/0.249	0.197/0.223	0.178/0.222
no. atoms			
protein	6982	3534	2496
ions/buffer	40	32	46
water	172	216	100
residual B factors			
protein	59	41	43
ligand/ion	73	48	69
water	44	40	56
r.m.s. deviations			
bond lengths (Å)	0.003	0.0059	0.0143
bond angles (°)	1.241	1.403	2.078
no. residues (%)			
favoured	427 (96.39%)	427 (96.39%)	304 (96.20%)
allowed	14 (3.16%)	14 (3.16%)	11 (3.48%)
high energy	2 (0.25%)	2 (0.45%)	1 (0.32%)

Data were collected from a single crystal for each structure. Three datasets were collected and merged from a single crystal for the B5-5 WuhRBD structure.

^a^ Values in parentheses are for the highest resolution shell.

In the H6–RBD complex, antigen binding involves exclusively CDR3, with additional individual contact residues in Framework 3 (figure 3*d*; electronic supplementary material, figure S6); for example, R54 in H6 forms a salt bridge with D420, which is conserved in all SARS-CoV-2 variants sequenced to date. Other key contacts between H6 and the RBD include hydrogen bonds between Y106 (H6) and D420 (RBD), and R54 (H6) with Y421 and N460 (RBD) (figure 3*d*; electronic supplementary material, figure S6).

The epitope recognized by A8 is located between RBD residues Y369 to K378 and binding is largely mediated through hydrogen bonding, with E113 in CDR3 of A8 forming a hydrogen bond network with S375, T376 and K378, and R56 in CDR2 with Y369, N370 and S371 (figure 3*e*; electronic supplementary material, figure S5). Both CDR2 and CDR3 of B5-5 make key interactions with RBD, notably salt bridges between R54 (B5-5) and D428 (RBD), and hydrogen bonding involving D428 (RBD) and Y55 in CDR2 and Y396 (RBD) with R99 in CDR3 (figure 3*f*; electronic supplementary material, figure S6).

**Figure 4.**
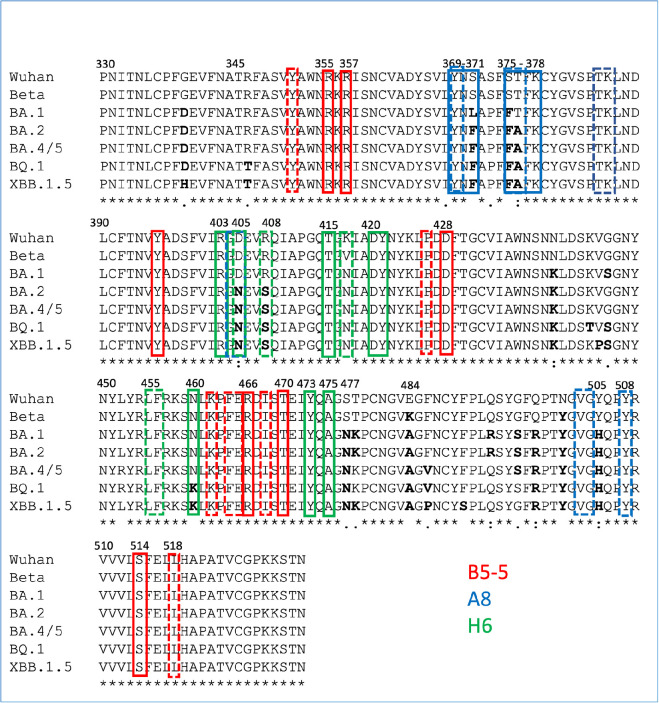
Alignment of the RBDs of SARS-CoV-2 variants annotated with key nanobody interacting residues determined by *in silico* analysis. Key RBD residues involved in nanobody binding (B5-5, A8 and A6) identified by *in silico* analysis of the respective crystal structures [31] are boxed in coloured boxes corresponding to the different nanobodies (B5-5, A8 and H6). Residues in bold are the mutations to the original Wuhan sequence found in the more recent Omicron variants.

Analysis of the key residues involved in the interaction between the nanobodies and RBDs provides a rationalization of the binding results to the Omicron variants. Thus, the epitope recognized by A8 in the Wuhan RBD structure is conserved in the sequence of the Beta variant but includes mutations in the BA.1 (S371L and S375F), BA.2 and BA.4/5 (S371F, S375F, D405N and R408S) subvariants, affecting hydrogen bonding (S371 and S375) and hydrophobic interactions (D405 and S408) (figure 4). Superimposition of the A8–Wuhan complex onto the structures of the Beta, BA.1, BA.4/5 from PDBs 8OWT (H6 structure), 7ZFB and 7ZXU, respectively, demonstrates the impact of mutations at residues 375 and 376 on A8 binding (electronic supplementary material, figure S7*c*,*d*). The S371F/L mutations are likely to be less detrimental and therefore have a reduced impact since hydrogen bonding to R59 of A8 occurs through the carbonyl of RBD residue at position 371 in the sequence, but this has not been confirmed experimentally.

The residues in the binding site recognized by H6 in the Beta variant are largely conserved in BA.4/5 and later variants, BQ.1, XBB, BA.2.75, though the mutation N460K in these later variants is likely to mean the loss of a hydrogen bond with R54 in H6 (figure 4; electronic supplementary material, figure S6). In BA.1 and BA.2, there are three other mutations in the Omicron RBD compared with the Beta and Wuhan that are located close to the H6 epitope, K/V417N, S477N and Y505H. Additionally, residue R408 is mutated to R408S in BA.2 and BA.4/5 but not in BA.1 (figure 4). None of these would be predicted to result in clashes or loss of key binding interactions with H6. However, there may be indirect effects of these changes. For example, the mutation at R408S would disrupt the non-covalent *pi*-interaction between H57 in H6 and R408 in the RBD (electronic supplementary material, figure S7*b*). Y505H interacts with R403 within the RBD which in turn does make important contacts with H6, the mutation could therefore alter the interaction made by R403. Collectively, these changes may partially account for the reduction in binding affinity to the Omicron BA.1 and BA.4/5 RBDs compared with the Beta RBD. The sequence of the epitope recognized by B5-5 is highly conserved among all SARS-CoV-2 variants, including Omicron BA.4/5 (figure 4) maintaining key interactions (electronic supplementary material, figure S7*e*,*f*). This is consistent with the observation that B5-5 binds to BA.1 and BA.4/5 with similar kinetics but does not account for the 10-fold reduction in affinity compared with binding to the Beta RBD (table 1). This suggests that there are more subtle structural differences between the RBDs of Omicron and Beta variants that modulate nanobody binding.

### 2.3. Inhibition of the binding of SARS-CoV-2 spike protein variants to human ACE-2 by trimeric nanobodies

We previously showed that assembling anti-SARS-CoV-2 RBD nanobodies into homotrimers, by joining the VHH domains with flexible Ser–Gly linkers, significantly increased binding because of avidity [1]. Therefore, trimeric versions of A8, H6, A10-5 and B5-5 were assembled and produced by transient expression in expi293 cells. The ability of the trimeric A8, H6, A10-5 and B5-5 nanobodies to block the binding of ACE-2 to SARS-CoV-2 spike trimer was determined in a multiplex competition assay in which activity against multiple spike protein variants was analysed in parallel. This is a commercial assay developed by Meso Scale Discovery (MSD) as a surrogate for viral neutralization testing [36]. Two sets of spike protein variants were evaluated, and the C1 and C5 trimeric nanobodies, previously shown to block ACE-2 binding, were included for reference [1]. The results confirmed that A8 and H6, but not B5-5 or A10-5, compete for the binding of ACE-2 to the spike but showed significant differences in inhibitory activity for the different variants (table 4; electronic supplementary material, figure S8). The H6 trimer inhibited ACE-2 binding to the spike protein across all the strains tested, including eight variants of the Omicron SARS-CoV-2 virus. By contrast, A8 was effective at blocking the ACE-2–spike interaction for Beta, Delta and Wuhan strains, but showed only partial inhibition of binding to BA.1 and BA.3 and no activity against the other Omicron subvariants. The inhibition pattern for C1 which shares a similar epitope to A8, was comparable to A8. As expected, C5 only inhibited ACE-2 binding to spike proteins that do not contain the mutations at E484 in the RBD (Wuhan, Alpha and Delta), the importance of the Arg–Phe–Glu interaction cluster has been previously noted [1,37]. As the most consistently active nanobody, H6 trimer was tested against further Omicron variants including XBB.1 and BQ.1, and showed similar inhibitory activity (table 5; electronic supplementary material, figure S8). However, there was no correlation between the affinity of the monomeric nanobodies to the isolated RBDs measured by BLI (table 1) and the potency of the trimeric versions in the MSD ACE-2 inhibition assay (table 4). For example, even though A8 and H6 monomers bind to BA.4/5 with similar affinities (*K*_D_ 8.1 and 7.08 nM, respectively), only the H6 trimer showed activity in the ACE-2 inhibition assay (table 4). This may reflect that they bind to different RBD sites and how these are presented on the immobilized spike protein in the ACE-2 inhibition assay.

**Table 4.** Inhibition (IC50) of ACE-2 binding to SARS-CoV-2 spike protein variants by trimeric nanobodies measured by electrochemiluminescence in MULTISPOT plates (Meso Scale Diagnostics).

variant	A8	C1	C5	H6	B5-5
**IC_50_ (nM**)					
ancestral	0.89	1.64	0.98	1.91	>100
Alpha	0.72	1.62	0.87	1.74	>100
Beta	0.8	1.84	NI	1.59	>100
Delta	0.67	1.94	0.97	2.08	>100
Omicron BA.1	10.87	3.26	NI	1.8	>100
Omicron BA.2.12.1	NI	>100	NI	3.39	ND
Omicron BA.2 + L452M	NI	>100	NI	4.28	ND
Omicron BA.2 + L452R	NI	>100	NI	3.3	ND
Omicron BA.2	NI	>100	NI	4.18	ND
Omicron BA.3	22.26	0.89	NI	2.39	ND
Omicron BA.4	>100	>100	NI	0.96	ND
Omicron BA.5	NI	>100	NI	1.09	ND

ND, Not Determined; NI, non-inhibitory.

**Table 5.** Inhibition (IC_50_) of ACE-2 binding to SARS-CoV-2 Omicron spike protein variants by H6 trimeric nanobodies measured by electrochemiluminescence in MULTISPOT plates (Meso Scale Diagnostics).

**v**ariant	BQ.1	BQ.1.1	XBB.1	BA.2.75	BA.2.75.2	BF.7	BA.4.6
IC_50_ (nM)	2.9	3	2.2	6.7	2.3	2.1	2.2

### 2.4. Neutralization of SARS-CoV-2 BA.5 *in vitro* by trimeric nanobodies

**Figure 5.**
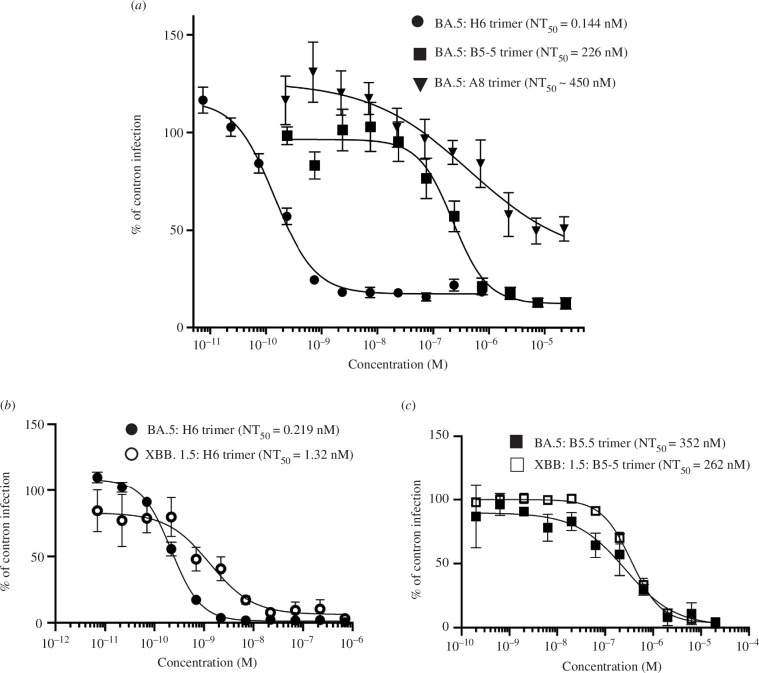
Neutralization of SARS-CoV-2 Omicron BA.5 and XBB.1.5 *in vitro.* Neutralization curves of the SARS-CoV-2 Omicron BA.5 and XBB.1.5 viruses by anti-RBD–nanobody trimers (A8, H6 and B5-5) measured in MNA. Data are shown as the mean (*n* = 4) ± 95% CI.

A micro-neutralization assay (MNA) was used to assess whether the activity of the trimers in the ACE-2 inhibition assay translated to the neutralization of live Omicron virus (BA.5). Although B5-5 did not compete with ACE-2 in the MSD and cell assays, it was included in the assays as there are precedents for monoclonal antibodies that do not inhibit ACE-2 binding that still show neutralization activity [38]. MNAs were first carried out with the BA.5 virus against which H6 was highly effective with an NT50 of 0.144 nM (figure 5*a*), whereas A8 showed only weak activity (figure 5*a*). In earlier studies, both H6 and A8 had shown equivalent neutralization activity against both Wuhan and Beta variants (electronic supplementary material, figure S9). Most interestingly, B5-5, which does not inhibit ACE-2 binding, did neutralize the BA.5 virus, though with a NT50 of 226 nM (figure 5*b*). Subsequently, H6 and B5-5 trimers were tested for neutralization of XBB.1.5 strain alongside BA.5. While the activity of B5-5 was similar (NT50 of 262 and 352 nM for XBB.1.5 and BA.5, respectively), H6 showed lower potency (NT50 1.32 nM for XBB.1.5 compared with 0.219 nM for BA.5) (figure 5*b*,*c*). There are several amino differences between the RBDs of XBB.1.5 and BA.5 (figure 4), including N460K in the H6 epitope that might account for the reduction in neutralization activity. However, the N460K mutation also occurs in the RBDs of XBB.1 and BQ.1. H6 competed equally well for ACE-2 binding to the spike proteins of XBB.1, BQ.1 and BA.5 in the MSD assay (tables 4 and 5). The only residue differences between the RBDs of XBB.1.5, XBB.1 (V486P) and BQ.1 (V486P and F490S) are outside of the H6 epitope. Further work will be required to explore whether these mutations cause structural changes in the spike protein that would modulate the interaction with H6. The neutralization potency of the H6 and B5-5 nanobody trimers was compared with Satrovimab, which is based on the human antibody, S309 [38], and retains activity against Omicron variants. The results showed that H6 trimers were more potent than Satrovimab in neutralizing XBB.1.5 viruses (NT50 of 7.1 nM versus 62.4 nM), while B5-5 trimers showed a similar activity compared with the reference monoclonal antibody (NT50 of 56.1 nM) (electronic supplementary material, figure S11).

The epitope recognized by B5-5 is conserved between the XBB.1.5 and BA.5 RBDS and insight into the potential mechanism of B5-5 neutralization comes from structural superpositions with the spike trimer (electronic supplementary material, figure S10). When the RBDs are in the down conformation, the B5-5 epitope is occluded by the S1-NTD of the adjacent spike monomer. With the RBD in the ‘up’ conformation, the cryptic epitope becomes accessible though binding of B5-5 would still impose a clash with the adjacent S1-NTD. It is therefore expected that neutralization by B5-5 arises from the destabilization of the spike trimer, as has been seen with other neutralizing nanobodies binding close to the B5-5 epitope [23,33].

### 2.5. Nanobodies provide preventive protection against SARS-CoV-2 Omicron infection of Syrian golden hamsters

**Figure 6.**
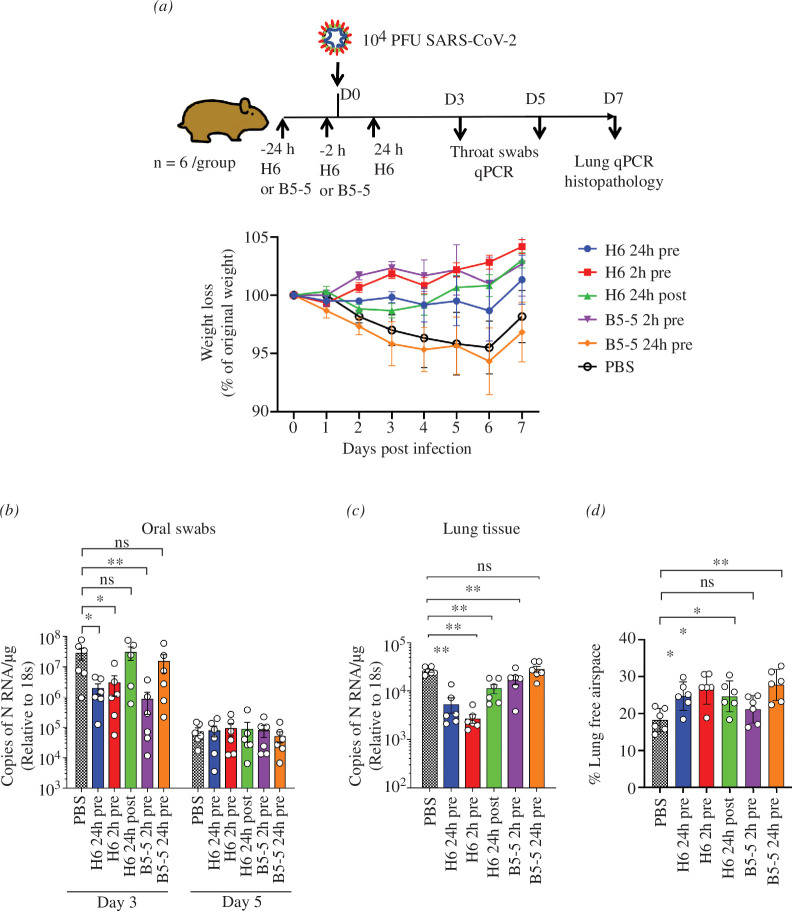
Prophylactic and therapeutic efficacy of nanobody trimers (H6 and B5-5) in the Syrian hamster model of COVID-19. (*a*) Golden Syrian hamsters (*n* = 6 per group) were infected intranasally (IN) with SARS-CoV-2 strain Omicron BA.5 (105 pfu in 100 µl PBS). Individual cohorts were treated either 2 h pre-infection, 24 h pre-infection or 24 h post-infection (hpi) with 2 mg kg^−1^ 100 μl of H6 or B5-5 IN as indicated or sham-infected with PBS. Animals were monitored for weight loss at indicated time points. Data represent the mean value ± s.e.m. (*b*,*c*) RNA extracted from oral swabs (*b*) and lung tissue (*c*) was analysed for SARS-CoV-2 viral load using qRT-PCR for the *n* gene levels by qRT-PCR. Assays were normalized relative to the levels of 18S RNA. Data for individual animals are shown with the median value represented by a horizontal line. Pairwise comparisons were made between groups using a Mann–Whitney *U*-test. **p < 0.01 and *p < 0.1. (*d*) Morphometric analysis. Haematoxylin–eosin (HE)-stained sections were scanned and analysed using the software program Visiopharm to quantify the area of non-aerated parenchyma and aerated parenchyma in relation to the total area. Results are expressed as the mean-free airspace in lung sections. Pairwise comparisons were made between groups using a Mann–Whitney *U*-test. *p < 0.05; **p < 0.01.

Based on the *in vitro* neutralization results, H6 and B5-5 trimers were selected for testing the efficacy of these nanobodies in the hamster model of COVID-19. The study design is shown in figure 5*a*, in which H6 trimers were administered either 2 or 24 h before or 24 h after challenge with SARS-CoV-2 Omicron BA.5, and B5-5 2 or 24 h before virus challenge. Omicron variants show reduced pathogenicity in the Syrian hamster model [39] compared with earlier strains reflected by a more modest weight loss by infected animals of 5% versus 15–20% observed in a previous study [1] (figure 6*a*). Nonetheless, either prophylactic or therapeutic treatment with the nanobody trimers prevented any weight loss in the animals infected with BA.5, except animals treated with B5-5 24 h pre-challenge (figure 6*a*). Both nanobodies were delivered via the nasal route and most interestingly administration of the H6 trimer 24 h before viral challenge was equally effective at preventing weight loss as the 2 h prophylactic treatment indicating that sufficient nanobody persisted in the airways 24 h after nasal instillation. By contrast, B5-5 was only effective when administered 2 h pre-challenge, possibly reflecting its lower potency compared with H6. Given its greater potency, only H6 was tested therapeutically by dosing 24 h after the virus challenge; it showed a reversal of initial weight loss by day 3 and returned to similar level as the other treatment groups by day 7 (figure 6*a*). Assessment of the viral RNA load in throat swabs and lung tissues by qRT-PCR showed that the prophylactic treatments with both H6 and B5-5 significantly reduced viral loads (approximately 1.5 log) in throat swabs on day 3 compared with the vehicle control, whereas there was no significant difference between the control and H6 animals treated post-challenge or B5-5 treated 24 h pre-challenge (figure 6*b*,*c*). By day 7, the viral loads in the throat swabs from all groups had reduced and were similar, whereas significantly lower levels of viral RNA were detected in the lungs of the animals pre-treated with H6 and B5-5 2 h before infection compared with the other treatment groups and phosphate-buffered saline (PBS) control (figure 6*b*,*c*).

**Figure 7.**
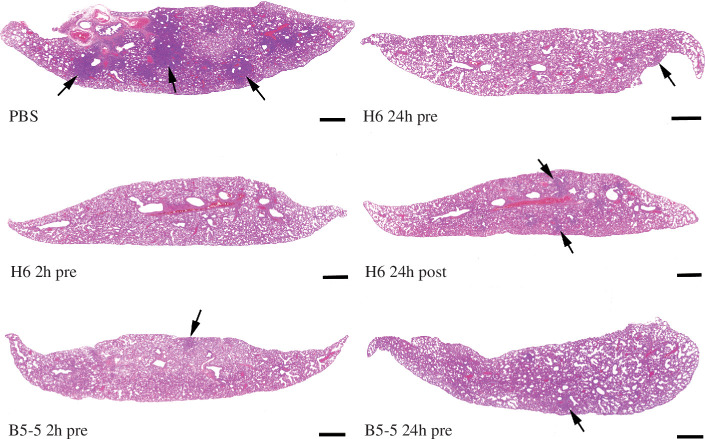
Lung histology. Lung sections of hamsters were infected IN with 10^4^ PFU per 100 μl SARS-CoV-2 and euthanized at day 7 post-infection. Animals had been untreated prior to infection (PBS) or treated with 2 mg/kg H6 IN 24 or 2 h pre-infection (H6 24 h pre and H6 2 h pre) or 24 h post-infection (H6 24 h post) or B5-5 24 h or 2 h pre-infection (B5-5 2 h pre and B5-5 24 h pre). In the untreated animal (PBS), the lung parenchyma exhibits large, consolidated areas (arrows), whereas in treated animals there are only a few small areas of consolidation (arrows). HE stain, bars = 10 µm.

The histological and immune-histological (detection of SARS-CoV-2 NP) examination of the control animals showed multifocal to coalescing consolidated areas (affecting 25–40%) with activated and hyperplastic type II cells, some de-squamed alveolar cells and an inflammatory infiltrate composed of macrophages, lymphocytes and neutrophils, accompanied by mild perivascular leukocyte infiltrations and periarterial oedema. Viral antigen expression was restricted to rare individual macrophages in consolidated areas, changes consistent with those reported in a previous study. Treatment with H6 reduced or completely inhibited the pathological changes. This was most obvious in animals that had received H6 2 h prior to infection. Here, the lungs appeared completely unaltered (4 hamsters) or showed minimal perivascular leukocyte infiltration (2 hamsters) and no viral antigen, suggesting that the lungs had not become infected at all. Two-thirds of the animals that had received H6 24 h pre-infection (*n* = 4) exhibited the typical consolidated areas with very limited viral antigen expression, but these affected only a small proportion of the tissue. In the remaining 2 hamsters, the lungs appeared basically unaltered. With H6 application 24 h post-infection half of the animals (*n* = 3) showed no lung alterations and no viral antigen expression, whereas the remaining 3 hamsters exhibited focal consolidated areas which in their extent were minimal (*n* = 2) or as extensive as in the control group (*n* = 1). Treatment with B5-5 had a less obvious effect than H6 when applied 2 h pre-infection. Five of the 6 hamsters exhibited the typical consolidated areas with very limited viral antigen expression, in the remaining animal the lung was unaltered. With B5-5 administration 24 h prior to infection, the effect was more obvious. While consolidated areas with very limited viral antigen expression were observed in 5 hamsters, their extent was minimal in 3; again, 1 animal showed an unaltered lung. Morphometric analysis of the lungs to automatically quantify the area occupied by ventilated and consolidated (i.e. non-ventilated) parenchyma confirmed that the extent of parenchymal consolidation was reduced in all treated groups; the difference was significant in all treated groups apart from the group that had received B5-5 2 h pre-infection (figures 6*d* and 7).

Similar efficacy results in a hamster model of COVID-19 have very recently been reported for a bi-specific antibody comprising two different nanobodies linked together and then fused to human IgG1Fc to create a bivalent molecule (designated 2-3-Fc). Treatment of animals infected with BA.1 either systemically or via nasal installation led to a reduction in viral load in the lungs of the hamsters, though no weight loss was observed in either treated or control groups which gained weight over the time course of the study [20] in contrast to our results following infection of hamsters with BA.5.

## 3. Discussion

The rapid emergence of the new Omicron strains of SARS-CoV-2 poses a significant challenge to the development of effective vaccines as immunity to earlier variants, including Omicron BA.1, does not prevent immune evasion from more recent strains, such as BA.4 and BA.5. This appears to be due to specific mutations in the RBD of the spike protein, notably L452R and F486V [6,40]. Similarly, many therapeutic monoclonal antibodies derived from patients infected with earlier strains of the virus are ineffective against the latest Omicron subvariants [7]. Antibodies that bind to epitopes that are highly conserved among closely related Sarbecoviruses, appear less susceptible to escape mutants than those that bind directly at or near the ACE-2 interaction surface. For example, the human monoclonal antibody S309, which binds to an epitope on the outer face of the RBD that includes the *N*-acetylglucosamine sugars attached to N343 [38], showed moderate losses (2- to 10-fold) in neutralization of Omicron subvariants BA.1, BA.2, BA.3 and BA.4/5 6,7,41–43].

The landscape of epitopes recognized by the large number of nanobodies that have been generated against the spike protein of SARS-CoV-2 mirrors that of human antibodies with the majority targeting the RBD [23,35]. By screening nanobody libraries from llamas immunized with the spike proteins of earlier SARS-CoV-2 strains (Wuhan and Beta), against the Omicron BA.1 spike protein, we have identified two nanobodies that neutralize the BA.5 variant in an animal model of COVID-19. The binding sites of these nanobodies, H6 and B5-5, have been mapped by determining the structures of nanobody–RBD complexes, identifying two neutralizing epitopes not specifically targeted by any other anti-SARS-CoV-2 nanobodies reported to date. These epitopes appear to be relatively conserved among Omicron subvariants, though escape mutants may still emerge, the locations of which are difficult to predict *a priori*. By systematically mutating individual residues in the RBD of the Wuhan sequence, Bloom *et al.* [44] identified residues that are mutationally constrained due to their roles in ACE-2 binding and/or folding of the RBD. Conversely, positions that may not be changed or may be more likely to mutate in circulating viruses, and therefore we examined the H6 and B5-5 epitopes in the light of the analysis by Bloom *et al*. [44]. For the H6 nanobody epitope, which has considerable overlap with the ACE-2 binding interface, the viable changes (folding and binding to ACE-2) within RBD at residues 405, 408, 415 416, 417, 421, 453, 456, 473, 474, 477, 487, 489 and 493 would not be expected to disrupt H6 binding. The mutation L455W and mutations that increase the size of the side chain of A475 (V, T, N and M) could result in clashes that disrupt binding to H6. The analysis shows that D420 tolerates many possible changes, and every change would result in the loss of a salt bridge with R54 of H6 nanobody. Moreover, changes that increase the size of the side chain relative to aspartic acid would be predicted to result in clashes with H6. However, mutation of D420 was calculated to be constrained due to the effects of changes on RBD expression (electronic supplementary material, table S1) [44]. The situation with B5-5 is rather different since it does not directly overlap with the ACE-binding site. Several residues within the epitope can be changed while preserving ACE-2 binding and folding. Residues R357 and R466 of the virus appears to tolerate multiple changes, all of which would alter hydrogen bonds, although none would be predicted to cause large van der Waals clashes. In contrast, the Y396W mutation would be predicted to delete hydrogen bonds as well as potentially causing van der Waals clashes with B5-5 nanobody. At D428, charge reversal mutation to lysine, which is plausible, could lead to electrostatic repulsion. However, D428 is predicted to be relatively invariant as negative effects on folding and expression were observed experimentally for mutations at this position (electronic supplementary material, table S1) [44]. The multiple possible mutations at T470 and S514 would disrupt a hydrogen bond and could, depending on the size of the new residue, introduce van der Waals clashes.

An obvious limitation of the above analysis is that the effects of mutations on protein conformation away from the site mutation cannot easily be predicted. Our work on the lab-derived nanobodies provided a demonstration of this, in which sequence changes in CDR3 resulted in increased affinity [37]. These changes did not alter the structure of CDR3 or create new contacts with the ACE-2, rather they resulted in structural changes in CDR1 which improved the interactions between ACE-2 and CDR2. Secondly, compensating additional mutations could rescue an apparently unviable single-site change. We observed a reduction in the *in vitro* neutralization activity of the H6 trimer for the XBB.1.5 Omicron variant compared with BA.5, which could not be readily explained in terms of the nanobody–RBD interaction.

In summary, we describe a small panel of nanobodies that bind the RBD of Omicron variants isolated from VHH libraries that we generated from llamas immunized with the spike protein of either the prototype virus or Beta variant, suggesting a strategy for identifying binders to any new SARS-CoV-2 variants. Two of the nanobodies (H6 and B5-5), configured as trimers, showed efficacy against Omicron BA.5 in the Syrian hamster model of COVID-19 with the nanobodies administered via the nasal route. The H6 trimer showed greater virus neutralization potency *in vitro* than Satrovimab, currently in use for the treatment of COVID-19 by intravenous administration; B5-5 trimer showed similar activity to the reference antibody. This suggests that these trimeric nanobodies either alone or in combination as bi-specific agents, may be useful starting points for the development of potential anti-viral immune-therapeutics, particularly given the precedent of an inhaled nanobody trimer for the treatment of respiratory syncytial virus (RSV) that progressed to early clinical evaluation [45].

## 4. Methods

### 4.1. Immunization and construction of VHH library

The SARS-CoV-2 Beta (B.1.351) trimeric spike protein (amino acids 1-1208) was produced [14] and antibodies were raised in a llama as previously described [1]. Briefly, spike protein (200 µg) was mixed with the adjuvant Gerbu LQ#3000 for each of the three intramuscular immunizations on days 0, 28 and 56. Blood (150 ml) was collected on day 66. Immunizations and handling of the llama were performed under the authority of the project licence PA1FB163A. VHHs were amplified by two rounds of PCR from cDNA prepared from peripheral blood monocytes and cloned into the SfiI sites of the phagemid vector pADL-23c (Antibody Design Laboratories, San Diego, CA, USA). Electro-competent *E. coli* TG1 cells (Agilent Technologies LDA UK) were transformed with the recombinant pADL-23c vectors, and the resulting TG1 library stock was infected with M13K07 helper phage to obtain a library of VHH-presenting phages.

### 4.2. Isolation of VHHs and construction of trimeric VHHs

Phage displaying VHHs specific for the RBDs of SARS-CoV-2 were enriched after two rounds of bio-panning on 50 and 5 nM of biotinylated RBD, respectively, through capturing with Dynabeads M-280 (Thermo Fisher Scientific). After the second round of panning, 93 individual phagemid clones were picked, VHH-displaying phage were recovered by infection with M13K07 helper phage and tested for RBD binding by ELISA with biotin-tagged RBDs immobilized on neutravidin-coated plates. For screening the Wuhan and Beta libraries with the Beta-RBD, an inhibition format was used to identify the highest affinity binders, in which soluble Beta-RBD was included in the assay as previously described [1]. Positive phage binders were sequenced and grouped according to CDR3 sequence identity using the IMGT/V-QUEST server [46]. Trimeric VHHs were constructed either as previously described by strand overlap PCR [1] or the completed sequence ordered as a gBlock (ITD technology). In the construction of the trimeric version of B5-5, residue Gly95 was changed to Asn. This mutation did not affect the binding affinity of the B5-5 monomer. The trimeric gene products were inserted into the pOPINTTGneo vector by Infusion cloning. pOPINTTGneo contains a mu-phosphatase leader sequence and C-terminal His_6_ tag [47].

### 4.3. Protein production

VHH plasmids were transformed into the WK6 *E. coli* strain and protein expression was induced by 1 mM IPTG during overnight growth at 28°C. Periplasmic extracts were prepared by osmotic shock and VHH proteins were purified by immobilized metal affinity chromatography (IMAC) using an automated protocol implemented on an ÄKTXpress followed by gel filtration using a Hiload 16/60 Superdex 75 or a Superdex 75 10/300 GL column, using PBS pH 7.4 buffer. The trimeric versions of the nanobodies were produced by transient expression in Expi293 cells and purified by a combination of IMAC and gel filtration in PBS pH 7.4 buffer. For animal studies, the final purified product was passed through two Proteus NoEndo clean-up columns (Generon, Slough, UK) to reduce endotoxin to <0.1 EU ml^−1^. Endotoxin levels were quantified using the Pierce LAL Chromogenic Endotoxin Quantitation Kit (Thermofisher Scientific). Protein was concentrated to 4 mg  ml^−1^ and flash frozen for storage at −80°C. Vectors encoding stabilized versions of the Beta and Omicron BA.1 trimeric spike proteins, containing twin proline substitutions and mutated furin cleavage sites, were generously provided by Piyada Supasa and Gavin Screaton (Nuffield Department of Medicine, University of Oxford, Oxford, UK) and the HexaPro BA.1 spike expression vector by Tiong Tan and Alain Townsend (Weatherall Institute of Molecular Medicine, University of Oxford, Oxford, UK). The Beta and BA.1 RBDs were amplified from the corresponding spike cDNAs and the BA.4/5 RBD was synthesized as a human codon optimized gBlock (IDT Technology). Biotinylated and non-biotinylated RBDs and spike proteins were expressed in Expi293 cells and purified, as previously described [14]. Satrovimab was from GlaxoSimthKline.

### 4.4. Biolayer interferometry affinity measurement of RBD binding nanobodies

Biolayer interferometry [25] was used to measure the binding constants of the nanobodies for various biotin-tagged RBDs immobilized on streptavidin sensors in 10 mM in 0.1% BSA (w/v) in 1× PBS, pH 7.4. All assays were performed in a black Greiner 96-well plate with a volume of 200 µl per well using a Sartorius Octet R8 system and designed using Octet BLI Discovery v. 12.2.2.20 software. For epitope binning, an association step (600 s) and a dissociation step (600 s) were performed with the first nanobody followed by an association step (600 s) and a dissociation step (600 s) with the second nanobody. Reduction of the *R*_max_ value of the second nanobody by 80% indicated competition for the same binding site as the first nanobody. Octet Analysis Studio v. 12.2.2.26 was used to analyse the data, with background normalization of the association and dissociation steps and Savitzky–Golay filtering. Curve fitting was applied using a global fit method and the association and dissociation rates were calculated using a best fit method. All graphs were plotted using GraphPad Prism.

### 4.5. MSD ACE-2 competition binding assay

All kit reagents were prepared as per the manufacturer’s instructions (Meso Scale Discovery, Rockville, MD, USA). A MULTI-SPOT 96-well, 10-spot plate was coated with multiple SARS-CoV-2 spike antigens (SARS-CoV-2 Plate 13 or SARS-CoV-2 Plates 23 and 27 was used). Assays were performed as per the manufacturer’s instructions. Trimeric nanobodies tested (A8, C1, C5, H6 and B5-5) were diluted to 0.001 mg ml^−1^ using the provided Assay Diluent, and serially diluted 1 : 3 down a separate dilution plate. Negative control (buffer only) and an internal control (calibration antibody known to block ACE-2) were included in each assay. All graphs were plotted with GraphPad Prism.

### 4.6. Binding of Omicron spike with ACE-2-GFP expressing cell line

An ACE-2-GFP constitutively expressing Calu3 cells were seeded in 8-well microscope slides (IBIDI GmBH) at 25 000 cells per well. The cells needed at least 24 h to adhere to microscope slide. After 36 h, culture media was removed and 300 µl of phenol red-free Dulbecco’s Modified Eagles Medium (DMEM)/F-12 media was added with fluorescently labelled BA.1 spike protein either with or without nanobody. The concentration of BA.1 spike protein was kept constant at 2 μM throughout the experiments, while nanobody concentration was varied. The cells were kept in the incubator for 15 min and colocalization of BA.1 spike with ACE-2-GFP was imaged using confocal microscopy. The real-time interaction between BA.1 spike and ACE-2-GFP was imaged at different nanobody concentrations and images were analysed using the ImageJ software. Corrected total cell fluorescence was calculated using the formula: corrected total cell fluorescence (CTCF) = integrated density − (area of selected cell × mean fluorescence of background readings).

### 4.7. Cryo-EM structures

Preparation of cryo-EM grids, data collection and processing were carried out as previously described [14]. Briefly, purified Wuhan spike-BAP protein in 10 mM Hepes, pH 8, 150 mM NaCl or super-stabilized Omicron spike BA.1 was incubated with nanobody H6, purified in PBS, at a molar ratio of 1 : 1.2 (spike monomer : nanobody) at 16°C overnight. Spike protein was used at a final concentration of 1 mg ml^−1^. The protein complex was centrifuged at 21 000 g, 16°C prior to grid preparation. Quantifoil 200 mesh R1.2/1.3 grids were glow discharged (Quorum) at 30 mA for 30 s, samples were applied to grids and plunged using a Vitrobot (Thermo Fisher Scientific). Grids containing stabilized Omicron Spike with nanobody H6 were screened, and data were collected on a Glacios microscope (Thermo Fisher Scientific), equipped with a Falcon IV detector, operated at 200 kV. Movies (40 frames each) were collected as gain reference-corrected files in counting mode using EPU (Thermo Fisher Scientific). For further data collection parameters, see electronic supplementary material, table S1. Processing of movies up to 2D classification was done automatically using the Relion_IT.py processing pipeline implemented at eBIC. In detail, motion correction and alignment of movies were performed using Relion (v. 3.1) [48] with a 5 × 5 patch-based alignment. Contrast transfer function (CTF) estimation of full-frame non-weighted micrographs was performed using CtfFind (v. 4.1.14) and non-template-driven particle picking was then performed within crYOLO [49] followed by 2D classification. The best 2D classes clearly showing details consistent with the spike complex were selected for 3D reconstruction and further 3D classification. 3D classes with different conformations were selected and further refined separately, before CTF refinement and particle polishing within Relion.

Data processing and refinement statistics are given in electronic supplementary material, table S2. PDB model 7QO7 and the crystal structure of the Wuhan spike-nanobody H6 was rigid body fitted into the map using Chimera [50] followed by Coot [51]. Four residues linking the RBD to the remaining spike were removed and the RBD was superimposed with the fitted crystal structure RBD, with the chains re-joined in Coot [51]. Due to the limitations in the map resolution, only one round of real-space refinement was conducted in PHENIX [52] to result in a final model. Data and refinement statistics are shown in table 2.

### 4.8. Determination of the structure of VHH–RBD complexes by X-ray crystallography

Purified VHHs were mixed with de-glycosylated RBD at a molar ratio of 1.2 : 1, and the complex was purified by gel filtration as described by La Bas *et al.* [53]. The optimal conditions for crystallization of each complex were A8-RBD: 0.2 M NaCl, 0.1 M potassium citrate, pH 4.2, 20% w/v PEG 8000; H6-RBD: 30% w/v PEG 4000; B5-5-RBD: 0.1 M Tris, pH 8.5; and 30% v/v PEG smear low. The protein complexes were crystallized at 10, 28.5 and 5.5 mg ml^−1^, respectively. Crystals were grown at 20°C using the sitting drop vapour diffusion method, cryoprotected with 30% glycerol (RBD-H6 and RBD-B5-5) or 30% PEG 400 (RBD-A8), cryocooled in liquid nitrogen, and diffraction data collected and processed at the beamlines I03, I04 and I24 of Diamond Light Source, UK. The structures were solved by molecular replacement with Phaser [54] as implemented in the CCP4i2 software suite [55] using the individual components of a previous nanobody–RBD structure (PDB 7Z1C) as the search models. The resulting structures were manually built in Coot [51] and refined using REFMAC5 [56] RBD–nanobody interfaces were analysed using PISA [31]. Data processing and refinement statistics are given in table 3.

### 4.9. Microneutralization assay

VHH trimers were serially diluted into DMEM containing 1% (w/v) foetal bovine serum (FBS) in a 96-well plate. SARS-CoV-2 strains (B VIC01, B1.17 and B1.351) passage 4 (Vero 76) (9 × 10^4^ pfu ml^−1^) diluted 1:5 in DMEM–FBS were added to each well with media only as negative controls. After incubation for 30 min at 37°C, Vero cells (100 μl) were added to each well and the plates were incubated for 2 h at 37°C. Carboxymethyl cellulose (100 μl of 1.5% v/v) was then added to each well and the plates were incubated for a further 18–20 h at 37°C. Cells were fixed with paraformaldehyde (100 μl well^−1^ 4% v/v) for 30 min at RT and then stained for SARS-CoV-2 nucleoprotein using a human monoclonal antibody (EY2A). Bound antibody was detected by incubation with a goat anti-human IgG horseradish peroxidase (HRP) conjugate and following substrate addition imaged using an ELISPOT reader. The neutralization titre was defined as the titre of VHH trimer that reduced the Foci forming unit by 50% compared with the control wells.

### 4.10. Evaluation of trimer prophylactic and therapeutic efficacy in the Syrian golden hamster model

Male Syrian golden hamsters (8–10 weeks old) were purchased from Janvier Labs (Le Genest-Saint-Isle, France). Animals were maintained under SPF barrier conditions in individually ventilated cages. For virus infection, an Omicron BA.5 strain of SARS-CoV-2 was used, kindly provided by Prof Wendy Barclay (Imperial college, London, UK), isolated from the UK in June 2022 and sequence verified. Animals were randomly assigned into multiple cohorts of six animals. For SARS-CoV-2 infection, hamsters were anaesthetized lightly with isoflurane and inoculated IN with 100 µl containing 10^4^ PFU SARS-CoV-2 in PBS. Hamsters were treated with 100 µl via the IN route with either H6 or B5-5 trimers in PBS. Animals were sacrificed at day 7 after infection by an overdose of pentabarbitone. Tissues were removed immediately for downstream processing. From all animals, the left lung was fixed in 10% buffered formalin for 48 h and then stored in 70% ethanol until further processing. Two longitudinal sections were prepared and routinely paraffin wax was embedded. Consecutive sections (3–5 µm) were prepared and stained with HE for histological examination or subjected to immunohistological staining. Immunohistology was performed to detect SARS-CoV-2 antigen, using the HRP method and the following primary antibody: rabbit anti-SARS-CoV nucleocapsid protein (Rockland, 200-402-A50) as previously described [1].

For morphometric analysis, the HE-stained sections were scanned (NanoZoomer-2.0-HT; Hamamatsu, Hamamatsu City, Japan) and analysed using the software programme Visiopharm (Visiopharm 2020.08.1.8403; Visiopharm, Hoersholm, Denmark) to quantify the area of non-aerated parenchyma and aerated parenchyma in relation to the total area (= area occupied by lung parenchyma on two sections prepared from the left lung lobes) in the sections, as previously described [1].

## Data Availability

The coordinates and structure factors were deposited in the wwPDB with accession nos. A8-H3-RBD (8OWT), H6-F2-RBD (8OWV), B5-5-RBD (8OWW) spike H6 EM maps and models are deposited in the EMDB and wwPDB under accession codes, PDB: 8OYT EMDB: EMD-17295 2 and PDB: 8OYU EMDB: EMD-17296. Nanobody sequences are provided in the electronic supplementary material, table S2 [57].
